# Review of the United States universal varicella vaccination program: Herpes zoster incidence rates, cost-effectiveness, and vaccine efficacy based primarily on the Antelope Valley Varicella Active Surveillance Project data

**DOI:** 10.1016/j.vaccine.2012.05.050

**Published:** 2013-03-25

**Authors:** G.S. Goldman, P.G. King

**Affiliations:** a Independent Computer Scientist, P.O. Box 847, Pearblossom, CA 93553, United States; b Facility Automation Management Engineering (FAME) Systems, 33 Hoffman Avenue, Lake Hiawatha, NJ 07034, United States

**Keywords:** Universal varicella vaccination, Varicella, Chickenpox, Herpes zoster, Shingles, Varicella vaccine efficacy, Varicella vaccine cost-effectiveness, Herpes zoster incidence

## Abstract

In a cooperative agreement starting January 1995, prior to the FDA's licensure of the varicella vaccine on March 17, the Centers for Disease Control and Prevention (CDC) funded the Los Angeles Department of Health Services’ Antelope Valley Varicella Active Surveillance Project (AV-VASP). Since only varicella case reports were gathered, baseline incidence data for herpes zoster (HZ) or shingles was lacking. Varicella case reports decreased 72%, from 2834 in 1995 to 836 in 2000 at which time approximately 50% of children under 10 years of age had been vaccinated. Starting in 2000, HZ surveillance was added to the project. By 2002, notable increases in HZ incidence rates were reported among both children and adults with a prior history of natural varicella. However, CDC authorities still claimed that no increase in HZ had occurred in any US surveillance site. The basic assumptions inherent to the varicella cost–benefit analysis ignored the significance of exogenous boosting caused by those shedding wild-type VZV. Also ignored was the morbidity associated with even rare serious events following varicella vaccination as well as the morbidity from increasing cases of HZ among adults. Vaccine efficacy declined below 80% in 2001. By 2006, because 20% of vaccinees were experiencing breakthrough varicella and vaccine-induced protection was waning, the CDC recommended a booster dose for children and, in 2007, a shingles vaccination was approved for adults aged 60 years and older. In the prelicensure era, 95% of adults experienced natural chickenpox (usually as children)—these cases were usually benign and resulted in long-term immunity. Varicella vaccination is less effective than the natural immunity that existed in prevaccine communities. Universal varicella vaccination has not proven to be cost-effective as increased HZ morbidity has disproportionately offset cost savings associated with reductions in varicella disease. Universal varicella vaccination has failed to provide long-term protection from VZV disease.

## Highlights

► In 2000, varicella incidence dramatically declined to 70% of the prevaccine rate. ► HZ reports significantly increased among adults aged 20–69 years from 2000 to 2001. ► Children with a prior history of varicella demonstrated HZ rates similar to adults. ► By 2002, the efficacy of the varicella vaccination had declined well below 80%. ► HZ morbidity costs have exceeded the cost savings from varicella-disease reductions.

## 1 Introduction

The varicella-zoster virus (VZV) is a member of a family of viruses known as Alphaherpesvirinae [genus and species: *Varicellovirus human herpesvirus 3* (*HHV-3*)] that upon initial exposure causes varicella (chickenpox) as a primary infection. The initial infection is followed by a variable latency period, after which the lifelong VZV in the dorsal root ganglia can subsequently reactivate as herpes zoster (HZ), commonly known as shingles, a secondary infection. Following short-term safety and efficacy clinical trials in the US, the varicella vaccine, Merck's Varivax^®^, was licensed for use in children 12 months and older by the US Food and Drug Administration (FDA) on March 17, 1995. On July 12, 1996, the US Centers for Disease Control and Prevention (CDC) published the recommendations of its Advisory Committee on Immunization Practices (ACIP) for universal varicella vaccination of all healthy, susceptible children aged 12- to 18-months with a single 0.5-mL vaccine dose [1].

Prior to the initiation of the universal varicella vaccination program in the US, most public health officials assessing the cost–benefit of vaccination to protect against varicella were principally concerned with data pertaining to clinical chickenpox—largely ignoring the potential effects of this vaccine on the interrelated HZ epidemiology. In fact, three US project sites (Antelope Valley, California; Travis County, Texas; and West Philadelphia, Pennsylvania) were initially selected and funded by the CDC to only perform active surveillance for varicella in order to ascertain the effects of the varicella vaccine on the population. Unfortunately, this limiting decision meant that the important baseline incidence data for HZ would not be collected.

## 2 Methods

In 1995, The Los Angeles Department of Health Services (LADHS), Acute Communicable Disease Control (ACDC) unit entered into a cooperative agreement with the CDC to establish the Antelope Valley Varicella Active Surveillance Project (AV-VASP) which immediately began conducting surveillance for varicella. The Antelope Valley surveillance region consisted of approximately 35 communities, covering approximately 2000 square miles, located about 50 miles northeast of Los Angeles, California in the high-desert plains, with a stable community of 300,000 residents (60% of which were found in the two principal cities of Lancaster and Palmdale). Approximately 300 different reporting units, representing all the identifiable sources in the study region, submitted varicella case logs biweekly to the project. Each case log consisted of a listing of the varicella cases encountered by a given reporting unit. The reporting units comprised all known public and private schools and preschool/daycare centers with enrollments of 12 or more children and approximately 90% of the public health clinics, hospitals, private practice physicians, health maintenance organization (HMO) offices, correctional facilities, and large employers in the region.

With verbal permission of a parent/guardian, a structured telephone interview was conducted regarding each reported varicella case under the age of 20 years old. To minimize recall bias, these interviews were usually conducted with the caregiver within 4 weeks of the case report date. The interview provided detailed demographic (to assist in detecting duplicate case reports), clinical (e.g., temperature of high fever if applicable, list of any pre-existing conditions and/or medications, rating of severity of illness and characteristics of rash at the time of peak illness, duration of the rash, etc.), and health impact data (e.g., days the parent and/or student missed school or work). If other potential susceptible and exposed household members were identified, these were re-interviewed in another 4–6 weeks. The data from each interview was entered into a computer database designed by project staff and implemented by Gary S. Goldman, PhD (Goldman), the project's Research/Epidemiology Analyst from 1995 to November 2002.

In 2000, HZ was added to the surveillance and data was collected in the same manner as previously described for varicella. For HZ cases aged 20 years and over, only demographic information was collected. In 2003, Goldman transported the AV-VASP database, consisting of several hundred demographic and clinical variables for each case, to the CDC for their continued processing and analyses. Because of (a) minor differences in algorithms, and (b) slight differences and/or changes in handling verified and probable cases, varicella and HZ case counts reported in this review may differ slightly from those presented in AV-VASP published studies and annual project summaries to the CDC.

A case of varicella was defined as illness with acute onset of a diffuse papulovesicular rash without other known cause that was diagnosed and/or reported by a licensed health care provider, school nurse, or parent. A case of HZ was defined as a unilateral vesicular rash in a dermatomal distribution, diagnosed by a licensed healthcare provider. In 2004, verified HZ cases included all cases validated by medical record review in addition to the case interview.

Initially, varicella case reports decreased 80%, from 2934 in 1995 to 587 in 1999. Since varicella typically displays a 5-year annual cycle in the Antelope Valley with a peak seasonal trend occurring during early winter and late spring, the reductions in varicella in 1996 and 1997 occurred during a natural decline, following the 1995 peak in this cycle, and were not the result of any early impact of varicella vaccination on the study region. The reality was that during the six surveillance years, 1995–2000, varicella vaccination likely had begun to significantly impact the otherwise naturally decreasing incidence trend only during the latter 2 or 3 years. In 2000 the number of varicella case reports increased to 836, which was still a 72% decrease relative to a naturally occurring cyclical peak in wild-type varicella cases reported in 1995.

Prior to the start of surveillance year 2000, Goldman recommended that HZ-case data be added to the surveillance based on anecdotal reports from long-time public school nurses who reported observing cases of childhood HZ that they had previously rarely, if ever, encountered. The CDC accepted the renewal grant application that included the HZ proposal. Thus, starting in 2000, VASP performed active surveillance for both varicella and HZ. The largest HMO (Kaiser—serving an estimated 30% of the study population) began regularly reporting HZ cases in 2002 based on filtering International Classification of Disease, Ninth Revision (ICD-9) codes for HZ-associated patient visits. Increased surveillance for adult HZ with the inclusion of skilled nursing facilities, dermatology practices, and internal medicine practices was proposed starting in 2005. Thus, HZ counts were significantly under-reported in years prior to 2005.

The study results presented in this review are primarily from this Antelope Valley population, which experienced relatively high levels of varicella vaccine uptake/coverage. Because the surveillance was active (and not passive), AV-VASP managed to collect 100% of the varicella case logs biweekly from all of the reporting units participating in the AV-VASP [2]. Moreover, the surveillance was able to detect sensitive trends early in the universal varicella vaccination program because of four contributing factors: (1) the survey region was relatively isolated geographically with few residents seeking healthcare or attending schools outside the region, (2) the population was relatively stable, (3) there was no sampling (whereas, some sampling occurred in the other two CDC-funded sites), and (4) the existence of two ascertainment sources (schools and healthcare providers) allowed the use of capture–recapture statistical methods to derive ascertainment-corrected counts of varicella and HZ case reports.

The AV-VASP data collection was uninterrupted and surveillance activities remained relatively stable through 2002. However, the 302 adult HZ cases reported to AV-VASP in 2003, representing an 18% decrease from the 368 cases reported in 2002, was an artifact associated with the lack of the AV-VASPs sending a reminder via fax to each surveillance unit failing to submit a timely biweekly report (as had been previously done by an automated fax system implemented by the Research Analyst from 1995 through 2002). AV-VASP operations and reporting patterns were again temporarily affected when a new Project Director was installed in 2004 following the resignation of the initial Project Director.

## 3 Early-determined HZ incidence rates were censored

**Fig. 1:**
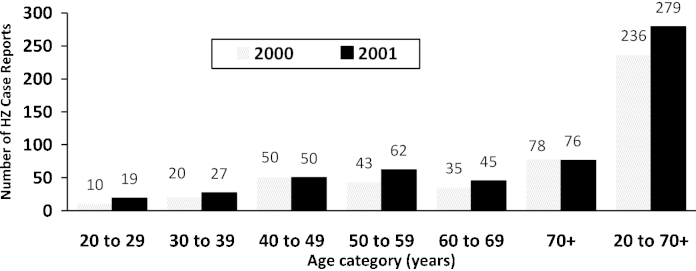
Number of adult HZ case reports by 10-year age category, Antelope Valley VASP, 2000 and 2001 [3,39].

By 2000, exogenous exposures to natural varicella (producing immunologic boosts) were dramatically reduced, especially after a marked decline in varicella incidence beginning in 1999 [2]. After 2 years of active HZ surveillance (2000 and 2001), Goldman noted that the number of HZ case reports had maintained or increased in every adult age category except elderly adults aged 70 years and older (Fig. 1) [3]. Using the paired *t*-test, the 28.5% increase in HZ case reports from 158 in 2000 to 203 in 2001 for ages 20–69 years was statistically significant (*p* < 0.042; *t* = 2.95, dF = 4) (Fig. 1). Also, the HZ incidence rate was low among vaccinated children under 10 years of age. However, the ascertainment-corrected, true HZ incidence rate of 307/100,000 person-years (p-y) during 2000–2001 [3] and 446/100,000 p-y during 2000–2003 [4] among children having a previous history of natural varicella exceeded that of any rates published in historical studies. Authorities within the CDC and AV-VASP were under the false impression that such vaccination-program related trends would take “15–20 years to manifest themselves”. They suggested that perhaps HZ incidence rates in the study population had always been unusually high, or were subject to greater diagnostic awareness, i.e., physicians were diagnosing more cases of HZ in the Antelope Valley region because AV-VASP was conducting active surveillance in that region. However, if this were true, AV-VASP would have expected to find that HZ incidence among vaccinated children was also unusually high and this was not the case. Consequently, the lower HZ rate in the vaccinated children and the expected rate in the 10–19 years age category negated the “unusually high” and “greater diagnostic awareness” hypotheses.

The next stated challenge was that the high HZ incidence rate reported among children with a prior history of varicella in the postvaccine period was due to bias associated with diagnostic error. However, since HZ was supposedly a rare disease in children (at least in the pre-licensure era), affecting 0.74 cases per 1000 children, it was unlikely that a physician would choose HZ as a differential diagnosis over more commonly occurring alternative diagnoses (e.g., herpes simplex virus—HSV, contact dermatitis, and impetigo).

While these observations were indeed preliminary and would require confirmation in other communities over a suitable longitudinal time period, AV-VASP and CDC opposed publication of these findings despite the reported HZ incidence rates having observation times (in terms of p-y) that were either similar to, or exceeded, that of other published studies by Guess et al. [5], Donahue et al. [6], Hope-Simpson [7], and Ragozzino [8].

After 6 years of varicella surveillance (1995–2000), the optimism of VASP/CDC was summarized as:

Varicella disease has declined dramatically in surveillance areas with moderate vaccine coverage. Continued implementation of existing vaccine policies should lead to further reductions of varicella disease in these communities and throughout the United States [2].

Yet, after 7 years (2000–2006) of HZ surveillance, the CDC would only report, “Data are inconclusive regarding an effect of the varicella vaccination program on herpes zoster epidemiology” [9]. A CDC study which found no increase in HZ incidence [10] was severely criticized since it was conducted in a population where varicella-vaccination coverage was not widespread in the community [11]. Two additional studies in 2011 reported no evidence of the universal varicella vaccination program contributing to increases in HZ in the US [12] and Canada [13]. But, both of these studies had serious methodological limitations including bias from not being conducted in a closed population since the data were acquired from a medical claims database and an administrative database, respectively—data from which individuals may disenroll causing their health history to cease to be tracked. Additionally, the Leung et al. study utilized US medical claims records from Marketscan^®^ databases with HZ ICD-9 codes which could not be validated through record reviews, though they were thought to be 85% or more accurate [12].

Thus, all the positive trends from the AV-VASP study data concerning single-dose vaccination for varicella, and, then, the deleterious trends that suggested the adoption of a two-dose varicella vaccination program (because of the observed waning immunity in a population with widespread vaccination coverage and because an increasing number of vaccinees were experiencing breakthrough varicella), were published in a timely manner [14–30], usually co-authored by Dr. Jane F. Seward [who initially headed the Varicella Epidemiology and Surveillance Division (VESD) of the National Immunization Program (NIP), and later served as Deputy Director, Division of Viral Diseases in the National Center for Immunization and Respiratory Diseases (NCIRD), CDC, Atlanta, GA], Dr. Aisha O. Jumaan (NIP and Division of Viral Diseases in the NCIRD, CDC), and Dr. Laurene Mascola (co-principal Investigator AV-VASP, Los Angeles County Department of Health Services, Los Angeles, CA).

### 3.1 The AV-VASP opposed publication of unwelcome HZ data

In November 2002, Goldman resigned from AV-VASP to objectively publish the other half of the VASP findings regarding HZ incidence rates and the use of capture–recapture methodology to correct for under-reporting. While all trends promoting one-dose and then two-dose protocols were encouraged and readily finalized for presentation at various symposiums and/or published by AV-VASP, unwelcome adverse outcomes concerning relatively high and increasing HZ incidence in the Antelope Valley community with moderate vaccination coverage were seemingly censored. Three manuscripts that Goldman had authored and which had been given such “low priority” by AV-VASP and CDC that they were unlikely to ever receive a formal internal review, were submitted to the journal *Vaccine*. Goldman, as a courtesy, informed AV-VASP and CDC of his intent to publish these manuscripts. Shortly thereafter, Dr. Laurene Mascola of the ACDC Unit of LADHS induced the Los Angeles County Legal Department to demand, in a letter dated April 10, 2003, that Goldman “cease and desist publication” of these manuscripts in a medical journal.

Goldman secured the services of a knowledgeable attorney, who, after reviewing the facts, advised the Los Angeles County's legal counsel: “(a) if your client persists in its efforts to restrain his findings, (b) if his findings enhance the public health, safety, and welfare, (c) if by seeking to restrain him from imparting valuable information concerning the lack of safety and effectiveness of the pharmaceutical being reported upon, and (d) if the County of Los Angeles has in any way been enriched by its participation in any study the results of which it seeks to restrain in this manner or any other manner whatsoever,” then there would be follow-up litigation under both the State and Federal False Claims Acts.

The county dropped its opposition and the manuscripts were peer-reviewed and accepted for publication as three consecutive articles in the journal *Vaccine* in October 2003 [3,31,32].

### 3.2 AV-VASP and CDC finally confirm Goldman's preliminary HZ incidence rates

In 2004, AV-VASP and CDC took issue with Goldman's presentation of HZ data and authored a criticism [33] of the three studies published in *Vaccine* by Goldman. Goldman's rebuttal response demonstrated those criticisms to be invalid on every account [34]. Further, the additional analyses that Goldman had performed using updated VASP data from 2000 to 2003 were published in 2005/2006 in *The International Journal of Toxicology* [4,35].

Ironically, in 2009, VASP and CDC published updated estimates of HZ incidence rates derived from AV-VASP case reports from 2000 to 2006 [36]. The study methodology used by Civen et al. was similar to that shared by Goldman with the AV-VASP staff in 2002 and detailed in 2003 [3] and 2005 [4] publications. Moreover, there were virtually no differences between the unadjusted cumulative 2000–2003 HZ incidence rates reported by Goldman [4] and the unadjusted cumulative 2000–2006 HZ incidence rates jointly reported later by VASP and CDC (Table 1). The ascertainment-corrected HZ incidence rates reported by Goldman (discussed in more detail in Section 4) were approximately double the unadjusted rates since capture–recapture demonstrated that AV-VASP had a 50% reporting completeness of HZ cases (Table 1).

Since medical costs associated with VZV were 4–5 times higher for those reactivating with HZ compared to those contracting varicella, only modest increases in the number of HZ cases would be sufficient to outweigh any benefits caused by reductions in varicella incidence, morbidity, and mortality [37] (Table 2). The co-principal investigators of AV-VASP were well aware of this concern. A supportive HZ-to-varicella cost ratio of 4.5 was reported in a Canadian province based on an analysis of only the direct costs during 1992–1996 [38].

### 3.3 Baseline crude and true HZ incidence rates in the Antelope Valley region

In 2000, the CDC had commissioned the AV-VASP to conduct a special survey among students (principally aged 10–14 years) in public middle (7/8th grade) schools within the Antelope Valley study population to determine the percentage of students susceptible to varicella. Interestingly, the susceptibility results of this Antelope Valley adolescent study closely mirrored those of another study population in Kentucky [31].

This adolescent survey was modified by Goldman (with the approval of the co-principal investigators) to collect information as to whether the student had had shingles (HZ) and, if so, at what age. By ignoring any HZ cases reported as occurring in the most recent 5 years (1996–2000) on the 4216 returned questionnaires, it was possible to derive a cumulative incidence rate of HZ among children who were under 10 years of age during the period prior to the start of the universal varicella vaccination program. The cumulative 1987-to-1995 crude HZ incidence rate among all children (including those still susceptible to varicella) was 72 cases/100,000 p-y which closely agreed with the crude rate of 68 cases/100,000 p-y reported by Guess et al. [5] and the crude rate of 74 cases/100,000 p-y reported by Hope-Simpson in Cirenchester, England [7]. The true incidence rate of HZ was roughly double, or 145 (95% C.I. 86–228) cases/100,000 p-y (18 cases/12,457 p-y) among only those children with a previous history of varicella—since about 50% of the children, principally aged 0–4 years, were still susceptible to varicella infection and hence, not candidates for HZ reactivation. Again, this derived true HZ rate was similar to the HZ rate of 133/100,000 person years aged <14 years in the population-based result reported in 1995 by Donahue in a Harvard Community Health Plan study [6].

These findings provided a quantitative rebuttal response to AV-VASP co-principal investigators’ challenge that, historically, perhaps the HZ rate among children in the Antelope Valley region had always been unusually high relative to children in other populations.

Based on the data collected from the adolescent surveys, Goldman wrote a paper detailing both the varicella susceptibility results and the baseline HZ incidence rates. The portion of the paper concerning varicella susceptibility was published virtually word for word with its authorship also attributed to the AV-VASP and CDC staff (Maupin T, Goldman G, Peterson C, Mascola L, Seward J, Jumaan A. Varicella susceptibility among adolescents in an active surveillance site. In: 36th National Immunization Conference of the CDC, Denver, CO; May 1, 2002). However, the AV-VASP project director demanded that the analysis and narrative pertaining to HZ be entirely deleted. The HZ incidence results would also be absent from the VASP's annual report—never to be discussed or considered in a separate publication. Shortly after leaving AV-VASP, Goldman published the adolescent study data and results—presenting both the number of adolescents still susceptible to varicella and the Antelope Valley region's baseline HZ incidence rates prior to widespread varicella vaccination [3].

### 3.4 Recurrent HZ incidence rate

The recurrent HZ incidence rate (i.e., the rate among individuals that reported having a second case of HZ that occurred six or more months following a first case) in the Antelope Valley study region was computed by dividing the number of reported second cases of HZ by the observation time that accumulated from the number of first-time-reported HZ cases. From January 2000 through April 2002 (2 years and 4 months) the recurrent HZ incidence rate in the AV-VASP was 2440/100,000 p-y (95% C.I. 1220–4374) based on 11 HZ recurrences during an observation time of 450 p-y. AV-VASP did not permit follow-up of the 11 recurrent HZ cases among adults to ascertain if they had pre-existing medical conditions or were immunocompromised. This post-vaccine recurrent HZ incidence rate was 3.3-fold (95% C.I. 1.0–10.3) higher than that of the pre-licensure era rate reported by the 2-year Harvard Community Health Plan study by Donahue et al. of 744/100,000 p-y (95% C.I. 203–1907) based on 4 recurrent HZ cases (3 of which were individuals that were HIV positive) during an observation time of 538 p-y [6].

### 3.5 Mounting evidence from preliminary HZ surveillance conducted by AV-VASP during 2000–2002 that supported the significant role of periodic exogenous exposures

Certainly, because of the relatively low observation time, the confidence interval was wide with respect to the recurrent HZ incidence rate. Given the small number of recurrent HZ case reports and low observation time, this wide confidence interval was not of major importance. However, the recurrent HZ incidence rate, being relatively high compared to a pre-vaccine rate, was one more factor contributing to the mounting evidence that individuals, who received periodic exogenous exposures from those shedding VZV, received significant subclinical boosts to cell-mediated immunity that helped to postpone or suppress their reactivation of HZ.

Thus, the mounting evidence came to include

• The high recurrent HZ incidence rate of 2440/100,000 p-y relative to the 744/100,000 p-y reported by Donahue et al. [6].
• The high HZ incidence rate among children under 10 years of age with a prior history of natural varicella of 307/100,000 p-y during 2000–2001 [3] and 446/100,000 p-y during 2000–2003 [4]—higher than any other historical rates.
• The 18% overall increase in adult HZ from 2000 to 2001; case counts maintained or increased in every age category except elderly adults 70+ years (Fig. 1); the 28.5% increase in HZ reports among adults 20–59 years was statistically significant (*p* < 0.042).
• The 56% increase in HZ case reports among adults 20 years of age and older from 236 in 2000 to 368 in 2002 [39].
• Baseline prevaccine cumulative 1987–1995 true HZ incidence rate of 145/100,000 p-y among children under 10 years of age [31].

## 4 Capture–recapture ascertainment-correction

The incidence rate estimates presented in most epidemiologic studies are extremely poor, missing 10–90% of the cases, with a high degree of variation [40–43]. This “missing cases” reality is even true of active surveillance where the attempt is made to count every case via comprehensive data collection from nearly all possible contributing sources. Without correcting for under-reporting, the AV-VASP incidence rates for varicella (and HZ) would simply reflect the level of case reporting and the unadjusted counts of varicella (and HZ) would contribute to the calculation of incidence rates that would not be comparable to other studies—especially those having either much lower or much higher levels of case ascertainment.

### 4.1 The National Health Interview Survey (NHIS) criterion standard validated the accuracy of using two-source capture–recapture methods

Due to difficulty in interpreting the population incidence results when the data consists of report counts that are incomplete (since ascertainment and enumeration of cases via active surveillance is never 100%), the AV-VASP utilized capture–recapture methodology with the healthcare providers and the schools serving as two independent ascertainment sources. To determine whether or not the capture–recapture estimates were accurate and the various capture–recapture assumptions of independence, 100% data linkage, and a closed population were valid, the 1995 ascertainment-corrected incidence rates in the age categories 1–4, 5–9, 10–14, and 15–19 years of age were compared with the gold standard, the 1990–1994 NHIS annual varicella incidence rates in the US. The AV-VASP reporting completeness of varicella cases aged 1–19 years was estimated at 46%, yielding an ascertainment-corrected incidence rate of 50.9/1000. The 1990–1994 NHIS varicella incidence rate of 53.2/1000 in this same age group was 4.3% higher [32]. Accepting the fact that some local variation in varicella incidence might be expected, the ascertainment-corrected rates for the Antelope Valley reasonably approximated those national rates reported by NHIS. This agreement confirmed the validity of using this statistics-based approach to correct for under-reporting in the Antelope Valley surveillance region.

The ascertainment-corrected varicella incidence rates in the AV-VASP for age categories 1–4, 5–9, and 10–14 years of age were lower than the incidence rates reported by NHIS. This may be due to the fact that schools often require a healthcare provider's written notice to excuse an absent student; thereby, creating a positive dependence of school and healthcare provider ascertainment sources. Under this scenario, the capture–recapture estimates likely represented lower bound values of the true varicella incidence rates [44].

### 4.2 Importance of using capture–recapture to obtain ascertainment-corrected counts for HZ cases reported to AV-VASP

Because the same reporting units that reported varicella cases, also reported HZ cases, it would be logical to presume that reporting completeness for both diseases was similar. This indeed proved to be true as shown in the following quantitative capture–recapture assessment: Consider that during 2000–2001, among unvaccinated children and adolescents aged 5–19 years, the schools reported 54 HZ cases and the healthcare providers reported 91 cases of HZ. Of these 145-case reports, 19 were duplicates. Although all 54 cases that were reported by schools sought attention from healthcare providers participating in the active surveillance project, only 19 (35%) were reported to the VASP by healthcare providers. Capture–recapture methods estimated the reporting completeness was 50% (95% C.I. 34–65%) [4].

In Table 1, taking into account the 50% under-reporting of HZ cases, the ascertainment-corrected HZ incidence rates shown in the right-most column are double that of the corresponding unadjusted rates in the adjacent column. Interestingly, the ascertainment-corrected HZ incidence rate of 28/100,000 p-y among vaccinated children aged 1–9 years (Table 1) compares with the rate reported by Tseng et al. of 27.4/100,000 p-y (95% C.I. 22.7–32.7) based on 172,163 vaccinated children with overall follow-up of 446,027 p-y, among children less than or equal to 12 years of age [45].

In 2003/2004, at two different conference presentations, Civen et al. reported the crude cumulative (2000–2003) HZ incidence rates using unadjusted counts of HZ cases reported to the AV-VASP making the incorrect assumption that AV-VASP had achieved 100% case ascertainment [46,47]. Thus, the reported post-licensure crude incidence rates of 40/100,000 p-y among children aged <10 years (of which the large majority had been vaccinated) and 45/100,000 p-y among individuals aged 10–19 years [47] seemingly lead to the conclusion that HZ incidence in the AV-VASP study population was low and there was negligible difference between HZ incidence rates among vaccinated and unvaccinated cohorts. As previously noted, the failure of the AV-VASP study to perform ascertainment-correction for the HZ case-report counts as was done in the Goldman study [4], precluded valid comparisons of HZ incidence rates reported in other studies having higher reporting completeness. McCarty et al. “strongly urge that all rates be reported only after formal evaluation and adjustments for under-ascertainment have been completed” [42].

Furthermore, the HZ incidence rates widely differed among vaccinated and unvaccinated cohorts of children aged 1–9 years. Therefore, AV-VASPs reporting a single mean HZ incidence of a bimodal distribution in this age category was statistically invalid and concealed the reality that the HZ incidence rate among children with a previous history of varicella was significantly higher than (a) incidence rates reported in other historical studies as well as (b) the incidence rate reported in the next 10- to 19-year age category.

## 5 Periodic exogenous exposures provided subclinical boosts to immunity

Prior to and during the early years of the universal varicella vaccination program, a controversy existed as to whether periodic exogenous exposures caused an immunologic boost that helped to suppress or postpone the reactivation of HZ in those children and adults that previously had a history of natural (wild-type) varicella [48,49]. Historical HZ incidence studies reported trends of HZ rates that increased with age.

In 1965, Dr. Hope-Simpson was first to suggest, “The peculiar age distribution of zoster may in part reflect the frequency with which the different age groups encounter cases of varicella and because of the ensuing boost to their antibody protection have their attacks of zoster postponed” [7]. However, prior to 1999, only limited studies existed that supported this hypothesis. For example, in 1983, Arvin et al. noted a boost in cell-mediated immunity (CMI) in 71% of adults who were exposed to varicella patients in the family [50]. In 1995, Terada et al. reported that Japanese pediatricians aged 50–69 who received multiple VZV exposures, demonstrated HZ incidence rates one-half to one-eighth that of the general population [51]. Geshon et al. in 1996 showed an immunologic boost that reduced the risk of HZ among leukemic children by reexposure to VZV, either by vaccination or by close exposure to varicella [52]. A 1998 study by Solmon found that pediatricians who had a greater incidence of exposure to VZV had lower rates of HZ than psychiatrists who had the lowest VZV exposure rates [53]. More recent studies by Thomas et al. [54] and Salleras et al. [55] have also demonstrated that re-exposure to VZV via contacts with children was associated with reduction in the risk of HZ in adults.

A number of studies have noted paradoxically that women, who generally have greater contacts with children, exhibit higher HZ incidence than men. This relationship of a higher female-to-male rate ratio experiencing HZ has been observed in diverse age groups from children and adolescents to older adults. The existence of a true gender difference in response to the reactivation of VZV as HZ, does not negate this fact: Regardless of gender, in any age category, when the number of exogenous exposures is increased, HZ incidence decreased; conversely, in the absence of exogenous exposures HZ incidence increased.

As the Antelope Valley region (and likely other communities) began experiencing 70% or more declines in varicella cases relative to the pre-licensure era, exogenous exposures to natural varicella became rare and it became increasingly apparent that both the efficacy and the safety of varicella vaccination had been confounded and overestimated in earlier clinical trials where vaccinees had been immunologically boosted by periodic exogenous exposures to children with natural varicella—especially those studies conducted in years having peak varicella transmissions due to seasonal epidemics.

By 2006, because vaccinated children were increasingly experiencing breakthrough varicella and failure of vaccine-induced disease protection to last “indefinitely” [24], the CDCs ACIP ignored the obvious failure of the assumptions that had been used to justify the initial vaccination program and, instead of stopping the one-dose universal varicella vaccination program, recommended that children receive two varicella doses—“the first dose should be administered at age 12–15 months, and a newly recommended second dose should be administered at age 4–6 years” [56]. Further, modeling revealed the potential for two-dose coverage to cause an increasing shift of varicella to older-age children and adults, where morbidity and mortality are increased [57]. In an effort to partially counteract the negative effects of the now two-dose universal varicella vaccination program on the epidemiology of HZ in adults, a shingles vaccine (Merck's Zostavax^®^) was recommended for adults 60 years and older in 2007 [58].

## 6 HZ incidence rates increased during the universal varicella vaccination program

### 6.1 Models predict “major epidemic” of HZ

Brisson et al. report that exposure to varicella is highly protective against HZ. Modeling this VZV transmission for the US, the study concluded, “Mass varicella vaccination is expected to cause a major epidemic of herpes-zoster, affecting more than 50% of those aged 10–44 years at the introduction of vaccination” [59]. Brisson et al. continued:

Eliminating varicella in a country…the size of the U.S. (280 million) would prevent approximately 186 million cases of varicella and 5,000 deaths over 50 years. However, our model predicts that eliminating varicella transmission could generate an extra 21 million cases of herpes zoster resulting in 5,000 deaths [59].

Another age-structured transmission dynamic model for the UK reported, “Routine infant varicella vaccination is unlikely to be cost-effective and may produce an increase in overall morbidity” [60].

### 6.2 Studies in communities with moderate varicella vaccination coverage that reported increasing HZ medical costs and incidence

Since the introduction of the varicella (chickenpox) vaccine in the US, there has been the concern that diminishing exposure to natural varicella could cause reactivations of the latent VZV as HZ. Patel et al. report that the net hospitalization costs for complications of HZ have increased by more than $700 million annually by 2004 for adults 60 years and older [60]. While rates of varicella-related hospital discharges decreased, there was an increase in HZ-rated hospital discharges, disproportionately among older adults [61].

From 2000 to 2001, reported HZ cases aged 20 years and older increased 18% in the AV-VASP (Fig. 1) [39] and this compares to the 22.5% annual increase reported in Massachusetts by Yih et al. from 1999 to 2003 [62]. A large 34% increase in HZ reports to AV-VASP was noted from 2001 to 2002 when an HMO (Kaiser) began reporting to the AV-VASP. This may have contributed to the high mean increase in HZ reports of approximately 28%/year among adults 20 years and older from 2000 to 2002 [39]. However, the AV-VASP reported a similar increase of 28% during 2006–2007 among adults aged 50 years and older as well as a larger 38% increase in HZ incidence among adults aged 50–59 years [63]. From 2000 to 2006, the incidence among those aged 10–19 years approximately doubled [63].

Thus, two population-based studies, conducted in two different, stable communities where (1) there was moderate varicella vaccination coverage (with greater than 50% of children under 10 years of age vaccinated) and (2) dramatic 70% reductions in wild-type varicella incidence rates relative to the pre-licensure era rates, reported significant HZ increases 4–8 years after the start of the universal varicella vaccination program [39,62,63].

**Fig. 2:**
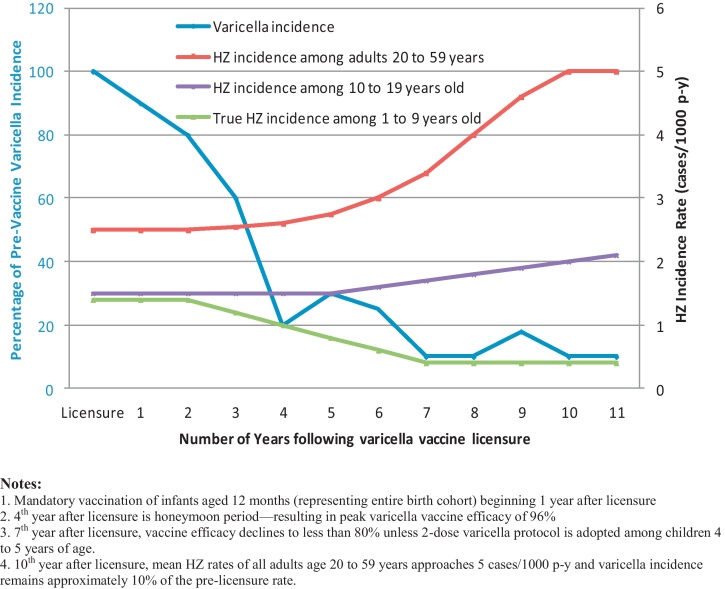
Relationships between varicella incidence and estimated HZ incidence in Antelope Valley community adopting universal varicella vaccination.

The inter-relationship of varicella incidence and expected HZ incidence rates is not very complicated as shown in Fig. 2. Ten years following licensure of varicella vaccine, varicella incidence (primarily in children, since adult varicella cases have only a negligible contribution) has been reduced to 10% of the prevaccine rate. A second varicella dose administered to children 4–5 years of age serves to sufficiently boost this cohort for the next 5 years such that varicella breakthrough disease and/or HZ reactivation stay in check at low levels among children—declining over time from the true pre-licensure HZ incidence rate of about 1.4 cases/1000 p-y to 0.4 cases/1000 p-y based on HZ reports to AV-VASP. The ascertainment-corrected HZ incidence rate among 10- to 19-year olds approximately doubled from 2000 to 2006 based on AV-VASP reports (Fig. 2). Ten years following licensure, virtually the entire cohort of children has been administered varicella vaccine which has boosted immunity that was previously provided in the prevaccine era by exogenous exposures to natural varicella. The curve depicting the adult (20–59 years) HZ incidence rate is estimated by considering (a) under-ascertained HZ reports to AV-VASP, (b) annual reported increases in adult HZ case reports, and (c) plausible limits of HZ incidence in the near absence of exogenous boosting. The adult HZ incidence rate increased over time from the pre-licensure HZ incidence rate of about 3/1000 p-y to 5/1000 p-y as exogenous exposures substantially diminished (Fig. 2).

Other studies with conflicting conclusions that reported no HZ increases [10,12,13] should not be hastily judged as contradictory when the underlying limitations in these studies are understood, e.g., (a) the study was conducted in a community having limited varicella vaccination coverage [10], and (b) data was extracted from a medical database that failed to track the health history of individuals that disenrolled and whose enrollment distribution by age differed from that of the true population [12,13]. Additionally, though the CDC has suggested that other long-term studies conducted prior to the start of universal varicella vaccination have demonstrated increasing HZ incidence trends, these studies (discussed in detail in Section 8) document much smaller annual percentage changes on the order of 1–3% which are attributed to studying an aging population.

Finally, it is constructive to consider additional reasons why several other studies conducted among adult populations reported low annual percentage increases in HZ incidence rates ranging from 5.6 to 9.1% [64–66] relative to the high annual rates reported by the AV-VASP [61,63] and Yih et al. [62] studies. The lowest annual percentage HZ increase, 5.6%, reported in Olmstead County, Minnesota between 1996 and 2001 [64], may be attributed, in part, to an aging population and minimal impact of varicella vaccination in those years. Minnesota did not require varicella vaccination for students entering kindergarten and 7th grade who lacked proof of having had chickenpox until 2004. Thus, the low uptake of varicella vaccine in Minnesota resulted in the incidence of natural varicella remaining high well beyond 2001. Annual percentage increases in HZ incidence among U.S. veterans reported by Rimland and Moanna [65] were lower than those found in Antelope Valley. It is likely that some veterans sought treatment at non-VA-facilities and some of those who were eligible for Medicare sought medical care in a private sector [65]. Furthermore, the age distribution of those participating in the military differs from that of the general U.S. population, preventing direct comparisons between the two cohorts [65,66]. Additionally, the increases in HZ incidence reported by Cockrill among individuals in the U.S. armed forces were also lower than those found in Antelope Valley. These rates may have also been influenced by the periodic administration of varicella booster vaccines as well as differences in occupational and leisure activities relative to those in the general population [66].

## 7 The CDCs “evidence” that HZ incidence rates had not increased

In 2002, Brisson et al., in a letter to the editor [67], criticized the CDC study that reported decreasing trends in varicella from 1995 to 2000 [2], stating, “…Seward had reported only *half* the story; shingles incidence rates should surely be reported alongside chickenpox incidence.” Seward's response to this deficiency was as follows:

*Because the population sizes in the varicella active surveillance sites are not sufficient to monitor age-specific herpes zoster incidence*, the CDC has funded two other sites—Massachusetts Department of Public Health and Group Health Cooperative (GHC) in Seattle—to conduct population-based varicella and herpes zoster surveillance and monitor age-specific incidence rates. Massachusetts has monitored incidence through a statewide telephone survey since 1998, while GHC is examining its automated medical records since 1992. *To date, no increase in herpes zoster is evident in any age group in either site (CDC, unpublished data, 2001)*. [*Italicization* added for emphasis.] [67].

### 7.1 The Massachusetts Department of Public Health Study lacked statistical power

The phone survey (not published in a peer-reviewed journal) conducted by the Massachusetts Department of Public Health included only 4916 and 3123 respondents reporting HZ cases among individuals aged 1–19 years in 1999 and 2000, respectively—for a total of 7319 p-y. During an overlapping 2-year period 2000–2001, the AV-VASP study region, comprised of 118,685 individuals in that same age group, accumulated a total observation time of 237,370 p-y, exceeding the Massachusetts study by more than 30-fold.

Notably, the varicella-vaccination coverage level in this Massachusetts study region was not as high as in the VASP study region. Thus, any increase in HZ incidence rates among the Massachusetts study population would be expected to be lower in magnitude relative to the VASP study region where approximately 50% of all children under 10 years of age had been administered the vaccine by 2000 [29]. Further, the small sample size of the Massachusetts study resulted in an unacceptably low statistical power to detect whether or not an increase in HZ incidence had occurred. Thus, the CDC knew, or should have known, that its conclusion that there were “no increases in herpes zoster incidence” based on this Massachusetts study was invalid.

### 7.2 The GHC study showed increasing trends in HZ incidence among adults and children during 2000–2002 when the varicella incidence rate dramatically declined

The GHC study had an annual average enrollment of 350,000 of all ages. It was similar to the 1995 population of 300,000 in the Antelope Valley region, yet Seward et al., in an author reply to Brisson et al., had indicated such population sizes were generally inadequate to monitor age-specific HZ incidence rates [67]. The GHC was promoted as having an observation time of more than 3.9 million p-y since varicella cases were tracked for 11 years, from 1992 to 2002, in the GHCs administrative health-records database [68]. The HZ incidence rates reported for the study population of all ages were statistically not significantly different in the pre- and post-varicella vaccination periods when age-adjusted to the US population in 2000 (an adjustment that was necessary because the number of persons in each age group enrolled during the study period was not constant).

The study reported that the age-adjusted HZ incidence rate declined 14.2% from a peak of 4.05/1000 p-y in 1992 to a nadir of 3.47/1000 p-y in 2000. However, based on the data plotted in that study's “Fig. 1”, HZ incidence rates began to rise—increasing 8% from the nadir in 2000 to about 3.75/1000 p-y in 2002 [68]. Interestingly, ignoring the unexplained high HZ incidence rate shown in 1992 and evaluating the trend from 1993 and 1994 to the same nadir point in 2000, the apparent HZ incidence rates decreased (approximately 11% and 6% [mean 8.5%], respectively) at a slower annual rate (1.8%/year and 0.86%/year, respectively) over 6–7 years relative to the faster 8% (4%/year) increase in HZ incidence rates during 2001 and 2002. Further, as the GHC study's authors admitted, the vaccination rates in Seattle, Washington were lower than the national average such that “few children [aged 0–9 years] had been vaccinated during 1996 and 1997” [68]. Yet, the reported age-specific HZ incidence rate for the GHCs enrollment population increased among both the vaccinated children (from “0” cases/100,000 p-y in 1998–1999 to “49” cases/100,000 p-y in 2002) and the unvaccinated children (increasing nearly 70% from “87”/100,000 p-y in 1996 to “145”/100,000 p-y in 2002) [68].

The serious population-sample limitation of the GHC study was confirmed by the reported 1992–1996 varicella incidence rates of 14.54, 8.2, and 1.9 cases/1000 among children aged 1- to 4-years, 5- to 9-years, and 10- to 19-years, respectively, which were only 14.5%, 9.9%, and 15.6% of the respective “gold standard” 1990–1994 rates reported by NHIS in these same age categories (Table 3). By contrast, the corresponding 1995 ascertainment-corrected incidence rates from AV-VASP data were 91.5%, 99.8%, and 89.3% of the reported NHIS incidence rates, respectively (Table 3) [32].

Thus, the GHC study was severely compromised by: (1) extreme under-reporting of cases (without the ability to perform ascertainment-correction), (2) a significantly lower level of vaccination coverage relative to the Antelope Valley communities under active surveillance, (3) variability associated with the enrollment in the records-tracking data system of the GHC, a Health Maintenance Organization (HMO), that was ever-changing over the 11-year study period, and (4) selection bias due to such factors as cost of enrollment and employers offering the HMO plan. Therefore, the conclusion that “the vaccination-associated decrease in varicella disease did not result in an increase in the incidence of HZ”, drawn from the GHC study data, was based on selective endpoint data that, given the preceding factors, was, at best, only somewhat representative of HZ incidence rates in the sample population enrolled and, therefore, could not be representative of true population trends for HZ incidence rates. Ironically, although not achieving statistical significance, the data suggest that, beginning in the post-vaccine year 2000, when the varicella incidence rate finally declined to less than 50% of the prior 1992–1999 mean rate, the age-adjusted HZ incidence rates (that included adults) increased through the 2002 study endpoint. Concomitantly, increasing HZ incidence rates were specifically noted by the study's authors among both vaccinated and unvaccinated children under 10 years of age.

### 7.3 CDC promoted two poorly designed studies

In view of the preceding facts, the CDC had funded two different studies (Massachusetts Department of Public Health and GHC) having fundamentally flawed methodologies with the apparent intent to dilute the AV-VASP findings and minimize the significance of Hope-Simpson's exogenous boosting hypothesis [7].

On September 2002, the 42nd Interscience Conference on Antimicrobial Agents and Chemotherapy (ICAAC) sponsored by *The American Society for Microbiology* was held in San Diego, California. During the Symposium on Varicella-Zoster Virus from 8:30 to 11:00 a.m., Dr. John Edmunds presented, “Potential Changes in Zoster Epidemiology with Childhood Immunization”. During the question phase, Dr. Jane Seward was acknowledged and stated categorically that in the U.S., at the various active surveillance sites, no increases had been seen in HZ incidence during the post-vaccination years—quoting statistics from both the Massachusetts Department of Public Health and GHC studies.

Later in 2002, Roche [69] refers to a personal communication from Dr. Jane Seward and writes: “Despite concerns of a rise in zoster, active surveillance for herpes zoster in the USA sentinel sites has not shown any change in herpes zoster incidence to date” [69]. Again, in a December 2002 Australian newsletter, Burgess writes, “There has been no change in the age-specific incidence of herpes zoster since the program started” [70]. Thus, CDC officials were not reporting the preliminary increases in HZ reported by the AV-VASP (Figs. 1 and 2).

In 2010, Australia also reported increases in HZ that started after the introduction of varicella vaccination [71,72]. Notably, though Varicella vaccination was recommended in Australia in 2000, only since September 2003 had the vaccine been publicly funded for use in all children at 18 months of age, which explained its delays in finding HZ-case increases.

## 8 Were increased HZ incidence rates among 10- to 19-year-olds comparable to increases found in pre-licensure studies?

Civen et al., in a VASP/CDC study presenting the cumulative incidence rate of HZ during 2000–2006 [37, “Table 1”], assumed that the AV-VASP had 100% enumeration of reported HZ cases. The AV-VASP/CDC authors explained that the 63% increase in HZ incidence rates, from 35 cases reported in 2000 (59.5/100,000 persons; 95% C.I. 42.7–82.9) to 64 reported in 2006 (96.7/100,000; 95% C.I. 75.7–123.6; *p* < 0.02) in the 10- to 19-year-olds [36] paralleled reported increases in other studies “even before varicella vaccine was available” [36]. Yet the 31% increase during 1979–1997 reported by Brisson et al. [73] represented only a 1.6% annual increase among adults—which was explainable in terms of the continually aging population. The approximate 35% increase in the HZ incidence rate in the study by Ragozzino et al. during 1944–1959 (16 years) similarly yielded about a 2.2% annual increase that was again typical of an aging population [8]. This in no way compared with the 63% increase during 7 years or 9% mean annual increase reported in an adolescent cohort by the AV-VASP.

## 9 Evidence in support of Dr. Hope-Simpson's 1965 hypothesis

In the Civen et al. study of HZ incidence, VASP/CDC authors conceded: “The possible reasons for this increased incidence [63% among those 10–19 years of age] cannot be confidently explained” [36].

To explain the high incidence in this cohort, consider the findings published by Hope-Simpson in which the crude HZ incidence rate reported in children aged <10 years is 74/100,000 p-y. This rate was slightly more than half that of the next age category, 10–19 years, or 138/100,000 p-y [7]. However, since approximately 50% of the children under 10 years of age were still susceptible to varicella and hence, not candidates for reactivation of HZ [31], the true HZ incidence rate among only those children with a prior history of varicella would have been approximately double the reported crude HZ incidence rate or about 148/100,000 p-y. Thus, the *true* HZ incidence rate was actually constant over the age range 0–19 years, being approximately the same in both age categories, 0–9 and 10–19 years. For the next three decades, those 20–49 years of age, the rate approximately doubled again to about 250/100,000 p-y, and then doubled again to about 500/100,000 p-y among adults aged 50–59 years [7].

Presuming Dr. Hope-Simpson's hypothesis was correct, as exogenous exposures diminished in an age category comprised of individuals with a previous history of varicella, those individuals would increasingly reactivate with HZ at an incidence rate approaching 500/100,000 p-y—the same rate currently found among adults with relatively few opportunities for immunologic boosting. Thus, the high HZ incidence rates in the post-licensure period, especially among children with a previous history of natural varicella, based on AV-VASP data [3,4,35] reported some 40 years after the 1965 Hope-Simpson paper, supported the hypothesis, “The peculiar age distribution of zoster may in part reflect the frequency with which the different age groups encounter cases of varicella” [7]. Additionally, unvaccinated children with a previous history of varicella may have greater sensitivity to exogenous exposures (boosting) and a poorer cell-mediated response following primary infection relative to older age groups. Thus, once adolescents and adults are exposed to VZV, they remain boosted for longer periods [4]. Table 1 indicates that the ascertainment-corrected rate of 446/100,000 p-y among children aged <10 years with a previous history of natural varicella had already approached the apparent rate limit observed in older adults.

Moreover, the study in Japanese pediatricians aged 50–69 years [50] demonstrated the Hope-Simpson hypothesis in the reverse direction—whereby the relatively high HZ incidence that characterizes adults was found to be as low as the pre-licensure rate in children or adolescents due to the physicians’ frequent exogenous exposures to children with natural varicella.

Of course, elderly adults both the pre- and post-licensure periods continue to experience a sharp increase in HZ incidence rates due to an age-related immune-system decline that begins at around 60 years and older [6].

## 10 Studies of the cost-effectiveness of the US universal varicella vaccination program

In 1994, Lieu et al. modeled the cost-effectiveness of a routine varicella vaccination program for US children. From a medical perspective alone, varicella vaccination was not cost effective since the annual vaccination costs of approximately $162 million exceeded the annual medical cost savings of $80 million (i.e., from the health payer perspective, $2 was spent for each $1 saved). However, by considering the cost of a parent's absence from work to care for a child with varicella, estimated at $392 million annually, single-dose varicella vaccination was now justified as being cost-effective from this societal perspective [74].

The preceding historical cost-effectiveness study was based on four key, but incorrect, assumptions: (1) vaccination cost is $35.00 per dose with only a $5.00 administration fee, (2) a single vaccine dose would confer life-long immunity, (3) vaccine efficacy would be high (85–95%) with negligible costs attributed to adverse effects associated with the varicella vaccine, and (4) universal varicella vaccination had no adverse effect on the occurrence of the closely related HZ in older individuals who had had natural varicella or on those who were administered the varicella vaccine.

The preceding assumptions all turned out to be seriously flawed. First, after FDA approval, the vaccine pricing has increased to nearly double the modeled cost. Moreover, as varicella vaccination coverage increased, a single vaccine dose was found to no longer confer long-term protection from either natural or vaccine-strain VZV infection. Additionally, HZ incidence rates were increasing in the near absence of the natural boosting that previously occurred in communities with annual varicella epidemics. For infants, the updated recommendation was that the first dose be given at age 12 months with a second (booster) dose given at age 4–6 years [56]. Based on these revisions to vaccine cost and number of doses alone, there was a fourfold increase in the annual cost of vaccination initially modeled by Lieu et al.—from $162 million to about $650 million—significantly offsetting the projected combined cost savings from both the medical and the societal perspectives.

Earlier, in 1999, Schuette and Hethcote developed a computer simulation model to evaluate the effects of a vaccination program for varicella using parameters estimated from epidemiological data. This simulation, which acknowledged discussions on varicella and HZ with John Glasser at the National Immunization Program CDC, concluded, “zoster incidence increases in the first three decades after initiation of a vaccination program” [75].

Prior to the licensure of a varicella vaccine, two UK university researchers, Garnett and Grenfell, had examined the implications of a universal varicella vaccination program and, in 1992, concluded, “Under some conditions, mass application of such vaccines may have the impact of increasing herpes zoster incidence…” [76].

Many updated cost–benefit analyses and studies of reduced morbidity and mortality focus on outcomes involving solely varicella—entirely ignoring the negative impact on HZ epidemiology [28,77,78]. Thus, both US and foreign studies still promote their flawed conclusion that such studies “confirm the positive impact of universal varicella vaccination” [79,80].

In 2005, Zhou of the National Immunization Program (NIP) performed an economic evaluation of the universal varicella vaccination program that included HZ in vaccinees and outbreak management costs, but excluded increasing HZ among those with a previous history of varicella and “potentially higher future post-vaccination incidence due to further accumulation of susceptible persons and future outbreaks” [81]. This study concluded, “compared to the one-dose program, the two-dose program may not be cost effective” [81].

By contacting the Elsevier life science editor, the CDC succeeded in having the journal *Vaccine* postpone a publication titled, “Cost–benefit analysis of varicella taking into account the closely related herpes-zoster epidemiology”, which was accepted October 4, 2003. This computer model, without even addressing the costs of serious adverse reactions to the varicella vaccine, reported that universal varicella vaccination had the impact of an additional 14.6 million HZ cases (or 42% increase) among adults aged <50 years during a 50-year period at a substantial medical cost burden of $4.1 billion or $80 million annually utilizing a very conservative estimated mean healthcare provider cost of $280 per HZ case [82]. After an attorney intervened, the paper was finally published and made available online on January 22, 2005.

## 11 Varicella vaccine efficacy declines rapidly after the “honeymoon” period

Vaccine efficacy refers to the effectiveness of a vaccine to prevent disease. In 1995, the CDCs ACIP claimed, “In clinical trials, the vaccine has proven to be effective for greater than 10 years in preventing varicella” [83]. Other reports suggested varicella vaccine confers long-lasting immunity of up to 20 years as shown in a Japanese study [84]. However, only 1 in 5 (20%) children were vaccinated in Japan so that incidence of natural varicella cases remained high, thus providing immunologic boosting to vaccinees when they contacted or were exposed to children shedding varicella [85]. In 1995, Merck & Co., the US vaccine's manufacturer, admitted that a boost in antibody levels had been observed in vaccinees following exposure to natural varicella, which could account for the apparent long-term persistence of antibody levels after vaccination and “the duration of protection from varicella obtained using Varivax in the absence of wild-type boosting is unknown” [86].

During the first years following licensure of the varicella vaccine, vaccine efficacy based on studies in clinical practices [87,88] and outbreaks in daycares and school settings [89–91] ranged from 70% to 100%. However, Galil et al. reported a 44% (95% C.I. 6.9–66.3%) effectiveness of the vaccine in a varicella outbreak among 25 (28%) of 88 children (in a daycare center between December 1, 2000, and January 11, 2001, having a high proportion of vaccinated individuals) [92]. Similarly, Lee et al. reported a 56% (95% C.I. 32.0–71.2%) effectiveness in an outbreak involving a primary breakthrough case followed by 54 cases in which 29 (53%) had been vaccinated in a Minnesota school with an enrollment of 319 students in 2004 [93].

When exogenous exposures (boosts) became rare, the vaccine was less effective and children soon needed a booster varicella vaccination. In 2002, Gershon suggested, “the time for exploring the possibility of routinely administering two doses of varicella vaccine to children seems to have arrived” [94]. In a 2004 study by the Epidemic Intelligence Service of the Centers for Disease Control and Prevention (CDC), the reported Varivax vaccine efficacy was 72% (95% C.I. 3–87%) [95]. Wide confidence intervals have characterized most outbreak investigations because of the relatively small numbers of varicella cases in children that are associated with any given local outbreak.

The AV-VASP investigated the secondary attack rate among household contacts aged 1–14 years during 1997–2001 [96]. That report states, “we analyzed the secondary attack rate by year; finding no trend, we conducted subsequent analyses for the 5 year period” [96]. That study's findings of a single mean efficacy of 78.9% (95% C.I. 69.7–85.3%) using contacts aged 1–14 years [96] concealed the discovery of a more than 20% decline in varicella vaccine efficacy by 2001 as the level of vaccination increased [4]. Although the 20% decline in vaccine efficacy from 1999 to 2001 was not statistically significant at the 95% confidence level (*z* = 1.96), it was significant at the 94% confidence level (*z* = 1.88). By July/August 2002, the varicella vaccine's one-dose efficacy had declined to 58.4% (95% C.I. 13.7–79.9%) which was statistically significantly lower than the peak 1999 efficacy [4] (Table 4). The one-dose vaccination program using Varivax demonstrated peak efficacy in 1999 (known as the “honeymoon” effect) because vaccinated children had just recently been boosted by the outbreaks of varicella that were occurring in the community at that time.

## 12 Safety of varicella and HZ vaccinations

### 12.1 Complications of introducing the vaccine- or Oka-strain varicella zoster virus (VZV)

Consider a child that is administered the live Oka-strain varicella vaccine and is subsequently exposed to an individual shedding VZV—either: (a) a child with varicella or HZ infection or (b) an adult with HZ infection. If the VZV strains are sufficiently heterologous (genetically distinct), a second case of varicella can result. There are at least five VZV genotype variations or virus clades known at this time, in addition to 4 rarely-reported provisional clades. Asymptomatic reinfection of a second strain can occur and establish latency, thus increasing the potential for HZ since both strains are subject to reactivation [97–99]. One study documents the transmission of a new VZV strain comprised of a marker exclusively characteristic of the Oka-strain in a wild-type isolate [100].

### 12.2 Reported adverse reactions and complications of live-virus varicella vaccination

Initial modeling of the cost effectiveness of universal varicella vaccination program assumed that vaccinated individuals would incur negligible adverse vaccine reactions and thus, vaccine-associated medical costs were not included in any of the models [74–82]. However, numerous published case studies document a wide range of deleterious outcomes in immunocompetent individuals following varicella (Oka-strain) vaccination of children or HZ vaccination of adults. Some of the adverse outcomes include ocular complications (e.g., blurred vision, herpes zoster ophthalmicus—HZO, visual loss or acute posterior multifocal pigment placoid epitheliopathy—APMPPE, interstitial keratitis, etc.) [101–108], central nervous system disease (e.g., encephalitis, acute disseminated encephalomyelitis—ADEM, acute cerebellar ataxia, VZV meningitis) [99,105,109–119], skin rash (e.g., urticaria, thrombocytopenic pupura, etc.) [120–123], HZ reactivation (including disseminated HZ and acyclovir-resistant HZ) [116,124–131], stroke following vaccination (varicella vasculopathy) [132], secondary transmission (to other children, adults, pregnant mothers) [133–136], pneumonia [137], varicella infection (i.e., breakthrough varicella) [138–140], decreasing immunity [141,142], Stevens–Johnson syndrome [143], fatal outcomes (e.g., child with acute lymphoblastic leukemia, fetal tissue calcifications and hydrops) [144,145], autoimmune disorders [142], and other miscellaneous reactions (e.g., reactions to gelatin, large local reactions, prolonged viremia, outbreak among school children that received 1- and 2-doses, and hematologic disease) [146–151].

Morbidity of even rare serious adverse events reported after varicella vaccination (some of which were enumerated in the preceding paragraph) contribute to offsetting the benefits of varicella vaccination [117]. In 2008, Chaves et al. reported that 15–20% of children who received one dose of varicella developed breakthrough varicella when exposed to VZV, some of which resulted in complications comparable to those occurring in unvaccinated individuals [26].

### 12.3 Anecdotal reports from parents reporting children experiencing multiple HZ cases

The following anecdotal reports from parents demonstrate the challenges associated with diagnosing and treating children experiencing multiple HZ reactivations. Interestingly, both US and foreign studies demonstrating reductions in varicella morbidity since the start of universal varicella vaccination [18,25,79,80] have excluded the analysis of rising morbidity from increasing cases of HZ. These studies may have inappropriately excluded this rising HZ-associated morbidity because the HZ medical costs far outweighed any projected savings from reduced varicella morbidity.

**Case 1**: “My daughter [‘*Sarah*’—name changed for confidentiality] has suffered from recurring childhood shingles since January 2003. Her last episode has just been resolved in early September 2004, because I was finally able to get her on the right antiviral medication with the proper dosage. *Sarah* was seen by two pediatricians, one allergist and two dermatologists, including one of the top pediatric dermatologists in our nation. Perhaps because childhood shingles is so rare, her case was misdiagnosed as atopic dermatitis. The treatment for this condition, as you know, includes baths to keep the skin moist and topical agents to keep it lubricated. This only worsened *Sarah*'s viral sores.”

“*Sarah* was prescribed 23 different oral and topical medications, including many doses of cortisone and antihistamines in 20 months. From the onset, I felt that my daughter's condition was systemic. It was only through my research and relentless questioning that lab-work was done. In July 2004, *Sarah*'s blood-work showed elevated levels of varicella-zoster virus. Although the physician she was working with at the time felt this was the result of her having chickenpox when she was two, I upon learning she could and should be re-tested within a month, requested this test. Her blood levels [of zoster] virus had nearly doubled.”

“…I am writing to you with the hope that you can help educate and advocate for this information to be made more available to the general public. As rare as childhood shingles is, by not catching this early on through the proper tests and the proper prescriptions for treatment, my daughter has suffered terribly. She now has post-herpetic neuralgia (PHN) as well…”

**Case 2**: On November 5, 2007, parents of a daughter with shingles reported: “…Our oldest daughter who is only 16 recently suffered from her second bout with shingles. She first had an episode of shingles at the age of 13. Our daughter NEVER had chickenpox, but was given the varicella vaccine in 1995. We were never told or even warned that it could cause shingles. We find it unbelievable that the ‘solution’ we are being provided is to go to the Infectious Disease Department at a local University Hospital in order to have them ‘help us manage’ this for the rest of our daughter's life. Now we have to remedy the shingles and we are altogether convinced that there will be many, many other young people adversely affected by what is a dangerous vaccine with awful side effects that stay with you for a lifetime…far worse than chickenpox in one's youth. Our daughter missed a week of school each time and suffered incredibly…”

**Case 3**: On September 22, 2008, a nurse contacted Goldman to share the following experience: “My son, who had natural chickenpox at 3 years of age, and who is now 16 years old, has been recovering for the past 6½ months from herpes zoster (with a rash in the T1 dermatome). He experienced vomiting and severe headaches that lead to a diagnosis of viral meningitis from central nervous system (CNS) complications of herpes zoster.”

Interestingly, the nurse indicated that the physician treating her son had encountered another teen with the same diagnosis a week prior to her son's case.

## 13 Costs to prevent HZ and post-herpetic neuralgia

In 2011, the FDA approved Merck's Zostavax^®^ for adults aged 50 years and older, but the CDC has, thus far, declined to recommend it for mass use. In late 2007, the CDCs ACIP recommended that the Zostavax vaccine, originally approved and licensed by the FDA in 2006, be given to all adults aged 60 years and older. Administering it is claimed to provide a boost to the adult immune system that helps to suppress or postpone the onset of HZ. However, natural boosts were previously available at no cost to adults from their periodic exposures to children who were actively shedding VZV in the community. Thus, the shingles vaccine raises the issue of not only achieving weak protective outcomes in adult vaccinations, but adults also tend to experience a higher rate of adverse vaccination effects relative to children, including a higher rate of serious adverse effects (possibly due to the 14-fold higher plaque forming units [pfu], or 19,000 pfu, relative to the varicella vaccine's 1400 pfu).

Based on the randomized, double-blind, placebo-controlled HZ trial by Oxman et al. which tracked 38,546 healthy subjects aged 60 years and older (median age 69 years) for a mean duration of 3.13 years [152] and using the current cost of about $200 per dose (based on the Rite Aid^®^ or Walgreens pharmacy fee), the *costs per year* to prevent one case of HZ and one case of PHN were, respectively: $35,000 (where number needed to vaccinate, NNV = 175) and $217,400 (where NNV = 1087) [153].

The placebo group (without exogenous boosting or those not administered the HZ vaccine) demonstrated an HZ incidence rate of 11.12 cases/1000 p-y, approximately 100% higher than the HZ incidence rate of 5.42 cases/1000 p-y among the cohort receiving the HZ vaccine [152]. Unfortunately, once the level of exogenous boosting diminished in the AV-VASP community via universal varicella vaccination, this same 100% increase in adult HZ incidence was observed in the surveillance data in the post-vaccine period relative to the adult HZ incidence in the pre-licensure era.

Interestingly, Brisson, using parameters obtained from the same Oxman study, assuming no waning of vaccine protection, and using a different, more conservative definition of NNV, estimates for adults 65 years and older that the NNV to prevent one case of HZ and one case of PHN *over their lifetime* is 11 and 43, respectively [154]. Using 20% per year waning of vaccine efficacy (average duration of 5 years), Brisson estimates a higher NNV of 41 (corresponding to $8200) and 229 (corresponding to $45,800), respectively [154].

## 14 Lack of understanding the human immune system

The human immune system is not fully understood—in fact man understands only the fringes of this complex system that involves much more than a balance between Th1 and Th2 responses in a system where there are more than 17 identified types of T cells (bone marrow immune-system cells [B cells] that mature in the thymus gland) [155,156].

Canniff et al. reported an association between those individuals with clinical or laboratory evidence of varicella-zoster virus (VZV) infection and lower risk of glioma [157], suggesting a protective effect of VZV against glioma [158,159]. The authors explained, “The protective effect of prior VZV infection against the incidence of glioma may be mediated by cytotoxic T lymphocytes (CTL) that recognize epitopes shared by VZV and glioma cells” [157].

In addition, Posner found that both the genetic background and previous immunological history of an individual play important roles in determining whether a person will be either: (a) protected from or (b) susceptible to adverse reactions caused by his or her exposure to viral antigens [160].

Further, Silverberg et al. also reported that wild-type VZV infection up to 8 years of age was found to be protective against atopic disorders that are thought to be “mediated by suppression of IgE production and allergic sensitization, as well as altered leukocyte distributions” [161].

Unfortunately, it is not possible to fully estimate the negative repercussions of the universal varicella vaccination program on such natural-chickenpox-protective outcomes as mentioned above.

## 15 Conclusion

Prior to the universal varicella vaccination program, 95% of adults experienced natural chickenpox [162] (usually as pre-school to early elementary school children)—these cases were usually benign. In the prelicensure era, the periodic exogenous boosting that adults received from those shedding VZV resulted in long-term immunity. This high percentage of seropositive individuals and their long-term immunity have been compromised by the universal varicella vaccination of children which provides at best 70–90% protection [142,163–166] that is temporary and of unknown duration—shifting chickenpox to a more vulnerable adult population which, as Dr. Jane Seward cautioned in 2007, carries 20 times more risk of death and 10–15 times more risk of hospitalization compared to chickenpox in children [167]. Thus, the proponents for universal varicella vaccination have failed to consider increased HZ-related morbidity as well as the adverse effects of both the varicella and HZ vaccines which have more than offset the limited benefits associated with reductions in varicella disease. The universal varicella (chickenpox) vaccination program now requires a booster vaccine for children and an HZ vaccine to boost protection in adults. However, these are less effective than the natural immunity that existed in communities prior to licensure of the varicella vaccine. Hence, rather than eliminating varicella in children as promised, routine vaccination against varicella has proven extremely costly [60,62,168] and has created continual cycles of treatment and disease.

## Figures and Tables

**Table 1:** Comparison of cumulative HZ incidence rates (cases per 100,000 p-y) derived by VASP/CDC (2000–2006) and Goldman (2000–2003).

Category (age in years)	Cumulative 2000–2006		Cumulative 2000–2003 HZ incidence rate [4]		Ascertainment-corrected HZ incidence rate^a^
	HZ incidence rate [36] (95% C.I.)	Sample size	Uncorrected HZ incidence rate (95% C.I.)	Sample size	
Vaccinated, 1–9	19 (15–25)	51	14 (9–21)	21	28
Natural Varicella, 1–9	239 (193–295)	84	223 (180–273)	94^b^	446
Natural Varicella 10–19	69 (61–77)	305	61 (51–72)	131	122

a The reporting completeness of HZ case reports was determined to be 50% (95% C.I. 34–65%) among those aged 5–19 years during 2000–2001.

b The number of shingles case reports included those verified (with interview conducted by phone) as well as those probable (reported by a physician, even though the case or case's parent/guardian could not be interviewed).

**Table 2:** Pre-licensure medical costs for herpes zoster are 4–5 times higher than the costs for varicella.

Description	Varicella (Chickenpox)	Herpes zoster (Shingles)
Number of cases	4 million^a^	1 million
Hospitalizations	11,000^a^	22,000^b^
Deaths	100^a^	400^b^
Medical costs	$275 million	$1.1 billion^c^

a Based on 5-year data prior to vaccine licensure.

b Miller E, Marshall R, Vurdien J. Epidemiology, outcome and control of varicella-zoster infection. Rev Med Microbiol 1993;4(October (4)):222–30.

c Yawn BP, Itzler RF, Wollan PC, Pellissier JM, Sy LS, Saddier P. Health care utilization and cost burden of herpes zoster in a community population. Mayo Clin Proc 2009;84(September (9)):787–94.

**Table 3:** Comparison of reported varicella incidence rates (cases/1000) by age category reported by the NHIS criterion standard with rates reported from GHC [68] and Antelope Valley VASP [32].

Age category (years)	GHC, Seattle, Washington 1992–1996		Antelope Valley VASP 1995		NHIS 1990–1994
	Varicella incidence rate	% of NHIS rate	Varicella incidence rate^a^	% of NHIS rate	Varicella incidence rate
1–4	14.53^b^	14.5	91.9	91.5	100.4
5–9	8.2^c^	9.9	82.7	99.8	82.9
10–19	1.9^c^	15.6	10.85	89.3	12.15
Mean % of NHIS rate	–	13.3	–	93.5	100.0

a Ascertainment-corrected incidence rates from Antelope Valley VASP by Goldman [32].

b Incidence rate given directly in GHC study results by Jumaan et al. [68].

c Incidence rate estimated from “Fig. 2” of GHC study by Jumaan et al. [68].

**Table 4:** Varicella vaccine efficacy among household contacts by year, 1997–2002, Antelope Valley VASP [4] and mean efficacy 1997–2002 [96].

Year of study	Vaccine efficacy stratified by year^a^ % (95% C.I.)	Mean vaccine efficacy^b^ % (95% C.I.)
1997	87 (75–93)	78.9 (69.7–85.3)
1998	94 (83–98)	
1999	96 (83–99)	
2000	86 (74–92)	
2001	74 (58–84)	
2002^c^	58 (14–80)	–

a Based on household contacts aged <20 years [4].

b Based on household contacts aged 1–14 years [96].

c Partial year of data collection through August 2002.
